# Enucleation of the Eyeball

**Published:** 1885-03

**Authors:** A. W. Calhoun

**Affiliations:** Professor of Diseases of the Eye Ear and Throat in the Atlanta Medical College, Atlanta, Ga.


### ENUCLEATION OF THE EYE-BALL.

### MORE EVIDENCE IN FAVOR OF HYDROCHLORATE OF COCAINE IN EYE SURGERY.

---

A CLINICAL LECTURE.

---

BY A. W. CALHOUN, M. D.,

PROFESSOR OF DISEASES OF THE EYE, EAR AND THROAT IN THE ATLANTA MEDICAL COLLEGE, ATLANTA, GA.

Reported for THE ATLANTA MEDICAL AND SURGICAL JOURNAL.

This patient, gentlemen, is suffering from sympathetic iritis of the right eye, as the result of injury to the left eye eight weeks since. You notice a considerable scar just below the temporal ridge and near the external angular process. This was caused by a knife wound. The knife blade penetrated the orbit and injured the ball to such an extent that all sight has been destroyed, and now we have marked sympathetic trouble in the other eye. In this case, as in all others of the kind, the safest thing to do is to enucleate the ball and thereby arrest the inflammation already set up in the right eye. If this is not done, the sound eye will soon be destroyed. It has heretofore been our custom to get the patient well under the influence of chloroform before beginning the operation, as it is a very painful one, but in this case we will try the effects of cocaine, the remedy you have so often seen us use in the clinics. We will vary the method of using it, however, in this case. In operations superficial in character, you know that all that has been necessary was to drop a few drops of the solution in the eye, but in this case we will, with the hypodermic syringe, inject five drops of a four per cent. solution into the tissues behind the ball. You notice there is almost complete anæsthesia of the conjunctiva now. This is due to a few drops of the solution which were put in the eye before the patient was brought in.

Now, with the needle introduced beneath the conjunctiva, just above the internal rectus muscle, we carry the point well back towards the apex of the orbit, and inject the fluid. For fear that this will not render the operation sufficiently painless, we introduce four drops on the opposite side of the ball, in the same manner just described.

The operation is made by grasping the conjunctiva with the fixation forceps and dividing it near the corneo-sclerotic junction the entire circumference of the ball, then the external and superior recti muscles are taken up and cut, after which the optic nerve and the remaining muscles are cut and the ball is removed with comparatively no pain. On examining the ball after removal, we find that the knife blade which caused the trouble penetrated the ball just above the attachment of the optic nerve. The fact that the entire tissues of the orbit are very much infiltrated and hardened from the inflammation resulting from the wound, renders the operation a little more tedious and difficult than these operations generally are.

While the operation has not been entirely painless, under the influence of the cocaine, it is far better to have used the cocaine than to have resorted to chloroform or ether; in the use of either of these agents there is more or less danger, and in cocaine there is absolutely no danger. The circulation of the patient has been slightly depressed, but this will soon subside by giving whisky. This depression from the use of cocaine is only observed after the use of it hypodermically.

